# Clinical Description of a Completed Outbreak of SARS in Vietnam, February–May, 2003

**DOI:** 10.3201/eid1002.030761

**Published:** 2004-02

**Authors:** Hoang Thu Vu, Katrin C. Leitmeyer, Dang Ha Le, Megge J. Miller, Quang Hien Nguyen, Timothy M. Uyeki, Mary G. Reynolds, Jesper Aagesen, Karl G. Nicholson, Quang Huy Vu, Huy Anh Bach, Aileen J. Plant

**Affiliations:** * Hanoi French Hospital, Hanoi, Vietnam; † Robert-Koch Institut, Berlin, Germany, and World Health Organization, Geneva, Switzerland; ‡ Bach Mai Hospital, Hanoi, Vietnam;; § Australian National University and Commonwealth Department of Health and Ageing, Canberra, Australia; ¶ Centers for Disease Control and Prevention, Atlanta, Georgia, USA; # Swedish Institute for Infectious Disease Control, Jonkoping, Sweden; ** Leicester Royal Infirmary, Leicester, UK; †† Hanoi Medical University, Hanoi Vietnam; ‡‡ Curtin University of Technology, Perth, Western Australia; 1 Members of the World Health Organization SARS Team in Vietnam.

**Keywords:** severe acute respiratory syndrome, SARS, coronavirus, clinical, Vietnam

## Abstract

We investigated the clinical manifestations and course of all probable severe acute respiratory syndrome (SARS) patients in the Vietnam outbreak. Probable SARS cases were defined by using the revised World Health Organization criteria. We systematically reviewed medical records and undertook descriptive statistical analyses. All 62 patients were hospitalized. On admission, the most prominent symptoms were malaise (82.3%) and fever (79.0%). Cough, chest pain, and shortness of breath were present in approximately one quarter of the patients; 79.0% had lymphopenia; 40.3% had thrombocytopenia; 19.4% had leukopenia; and 75.8% showed changes on chest radiograph. Fever developed on the first day of illness onset, and both respiratory symptoms and radiographic changes occurred on day 4. On average, maximal radiographic changes were observed on day 10, and fevers subsided by day 13. Symptoms on admission were nonspecific, although fever, malaise, and lymphopenia were common. The complications of SARS included invasive intubation and ventilation (11.3%) and death (9.7%).

The global outbreak of severe acute respiratory syndrome (SARS) has been epidemiologically linked to an outbreak that is believed to have begun during November 2002 in Guangdong Province, People’s Republic of China (*1*). SARS then spread to other countries and regions, such as the Hong Kong Special Administrative Region of China, Vietnam, Singapore, Canada, and Taiwan. By the end of the outbreak, 26 countries had reported 8,098 probable cases of SARS and 774 deaths (*2*).

Coronavirus was first hypothesized to be the etiologic agent of SARS by Peiris et al. (*3*). Later, two independent teams (*4*,*5*) confirmed the novel coronavirus was associated with SARS infections in patients from Hong Kong, Vietnam, Canada, and Taiwan. This article describes the clinical and laboratory features of patients with SARS in Hanoi, Vietnam.

## Methods

### Case Definition and Ascertainment

We used the World Health Organization (WHO) case definition (April 1 revision) for SARS in this investigation (*6*). A probable case-patient was defined as a person who sought treatment after November 1, 2002, with a high fever (>38°C) and cough or breathing difficulty and infiltrates shown on chest radiograph, consistent with pneumonia or respiratory distress syndrome. A probable case-patient was excluded if an alternative reason could fully explain the illness, e.g., proven tuberculosis or clinical response within 48 hours to antibacterial therapy. For practical purposes, we modified the case definition to only include cases occurring on or after the February 23, the date of onset of symptoms of the Vietnam index case. Serologic testing for SARS-associated coronavirus (SARS-CoV) was performed on serum specimens as previously described (*4*).

Case-patients were identified by clinicians, and considerable effort was made by the Vietnam Ministry of Health to train both metropolitan and rural staff in surveillance and identification of SARS. Many case-patients were admitted to hospital as having suspected SARS; however, only those whose condition conformed to the WHO case definition are included in this analysis.

The medical records of SARS case-patients were retrospectively reviewed by physicians. We used a standardized data collection form to record patient information. For the nine patients admitted to the hospital after March 20, clinical data were collected prospectively. For each case-patient, clinical signs, symptoms, radiologic findings, and data from biochemical, hematologic, and microbiologic tests throughout the course of illness were recorded. When assessing the proportion of case-patients with symptoms, if the information about a symptom was not recorded, we assumed the symptom did not occur. For the hematologic and biochemical course of illness, all available measurements were used, with recordings for >15 case-patients per day, and the measurements are displayed with accompanying standard deviation of means. Onset of illness was defined as the date when each case-patient first reported feeling unwell with symptoms compatible with SARS.

### Data Analysis

Data from the medical records were entered into Microsoft Excel and analyzed with Epi-Info version 6 software. We analyzed the data by using standard descriptive statistical techniques. To describe the course of the illness, the maximum temperature, leukocyte count, platelet count and lymphocyte count data from every case were combined and averaged for each day of the illness.

## Results

The first SARS case-patient in Vietnam was admitted to the hospital on February 26, 2003, and the last case-patient was admitted on April 8, 2003. All 62 patients with probable SARS were admitted to hospitals in Hanoi, Vietnam. The initial case-patients were admitted to a small private hospital (hospital A), and the later case-patients were admitted to a facility at a large public hospital, hospital B. Of the 62 case-patients, 61 (98.4%) were seropositive for SARS-CoV. The number of case-patients who were suspected of having SARS but later excluded is not known.

### Study Population

The mean age of SARS patients was 40.8 years (median 43, range 20–76 years) and 39 (62.9%) were female. A detailed description of the epidemiology of the SARS outbreak in Vietnam will be published separately.

### Clinical Features

#### Symptoms

**Figure 1:**
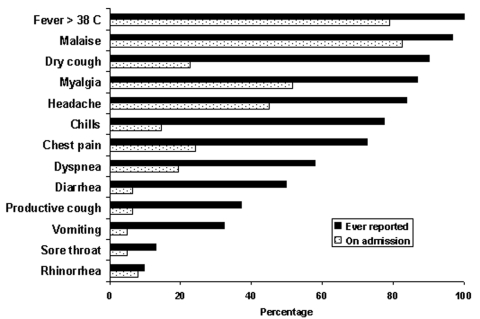
Symptoms of patients with probable severe acute respiratory syndrome (N = 62), at hospital admission and reported during the course of illness, Vietnam, February–May 2003. Note: All case-patients had fever during their illness because this was part of the case definition.

The most prominent symptoms on admission were malaise and myalgia (Figure 1). Less than one quarter of the patients had symptoms of the lower respiratory tract on admission; dry cough (22.6%), chest pain (24.2%), and dyspnea (19.4%). The proportion of patients who reported dry cough at any time throughout the illness increased to 90.3%. Other lower respiratory tract symptoms also became more prominent after admission. Upper respiratory tract symptoms were reported infrequently.

### Signs

Fever was present at admission for 79.0% of case-patients, with 66.1% having fever >38°C, although, as per the case definition, all case-patients experienced fever during their illness. Crepitations were present on admission in 35.5% of patients, and in 87.1%, crepitations developed during the course of their illness. On admission, 47 (75.8%) patients had abnormal chest radiographic results. The radiographs of the remaining 15 case-patients showed abnormalities 2 to 7 days (median 5) from the admission date.

On admission, the radiographic changes were mainly interstitial infiltrates, bilateral or unilateral, affecting less than two-thirds of the lungs. Maximal radiographic changes during the illness were mainly bilateral interstitial infiltrates or bilateral alveolar opacities affecting more than two-thirds of both lungs. The degree of change on the chest radiograph did not always appear to correlate with the apparent severity of illness as defined by the need for respiratory support.

The mean white blood cell count on admission was 5.9 × 10^9^/L, ranging between 2.7 and 16.3 × 10^9^/L (Table). Leukopenia was found in 19.4% of patients, and lymphopenia occurred in 79.3% of case-patients on admission, with lymphopenia defined as total lymphocyte count below 1.5 x 10^9^ /L. Thrombocytopenia was observed in 40.3% of patients on admission, with a mean platelet count of 160.7 × 10^9^/L.

**Table:** Hematologic and biochemical features of patients with severe acute respiratory syndrome (SARS) on admission

Parameter	Range		Mean	Median	Normal range	Abnormal		N
	Low	High				% Low	% High	
Leukocytes (× 10^9^/L)	2.7	16.3	5.9	5.3	4–10	19.4	6.5	62
Neutrophils (%)	44.0	92.8	70.7	71.0	40–75	-	37.1	62
Lymphocytes (%)	4.7	50.0	22.4	22.0	20-45	38.7	3.2	62
Lymphocyte count (× 10^9^/L)	0.3	2.2	1.1	1.1	>1.5	79.3	-	58
Hemoglobin (g/L)	88	216	132.4	132.0	125–155	25	1.7	60
Hematocrit (%)	0.273	0.468	0.388	0.386	0.40-0.52	25	1.7	60
Platelets (× 10^9^/L)	53	293	160.7	158.0	150–450	40.3	-	62
C-reactive protein (mg/L)	1	136	24.7	17.0	0-8	-	75	44
Alanine aminotransferase (UI/L) Hospital A Hospital B	8.0 13.0	36.0 294.0	22.3 70.0	22.0 49.0	10–50 <40	3.4	34.5	12 17
Aspartate aminotransferase (UI/L) Hospital A Hospital B	23.0 19.0	89.0 550.0	43.7 101.0	38.0 57.0	10–50 <37	–	42.9	11 17
Sodium (mmol/L)	129	148	137.1	138	135–145	29.6	3.7	27
Potassium (mmol/L)	3.3	4.7	3.9	3.9	3.5–5.0	14.8	–	27
Creatinine (mg/L)	48	133	93.2	93.0	5.6–12.4	4.5	9.1	22

Twenty-seven of the patients had biochemical blood tests performed. For these patients, 34.5% had elevated alanine aminotransferase levels, and 42.9% had abnormally high levels of aspartate aminotransferase. We observed hyponatremia in 29.6% of patients on admission, and 14.8% of patients had hypokalemia.

### Natural History of Illness

**Figure 2:**
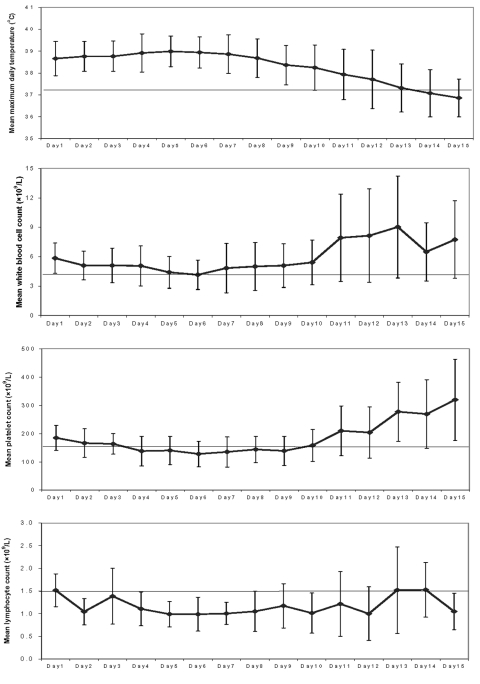
Average (±1 standard deviation) maximal daily temperature, leukocyte count, platelet count, and lymphocyte count by day of severe acute respiratory syndrome from onset, Vietnam, February–May 2003, (N = 62 cases but not for each data point).

The average maximum temperature for all of the case-patients on day 1 of onset was 38.7°C and reached a maximum of 39.0°C on day 5 (Figure 2). We observed that fever in SARS patients subsided on day 13. Overall, the average leukocyte count of all the cases never decreased below 4.0 × 10^9^/L, suggesting that leukopenia was not a common feature of SARS among the whole cohort, but did occur in a few patients, as indicated by the error bars on Figure 2. Thrombocytopenia (platelet count < 150 × 10^9^/L) was present in the cohort from day 4 until day 9 of the illness. After day 10, the average platelet count returned to within the normal range. Lymphopenia (lymphocyte count <1.5 × 10^9^/L) was present throughout the course of the illness, with lymphocyte counts ranging from 1.0 to 1.5 × 10^9^/L.

**Figure 3:**
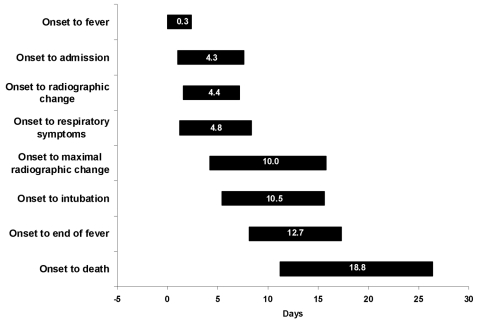
Average (±1 standard deviation) duration of time from onset of illness until outcome in the evolution of severe acute respiratory syndrome, Vietnam, February–May 2003

The natural history of SARS in Vietnam is shown in Figure 3. Not all patients felt feverish at onset, but fever developed an average of 0.3 days after the onset of other SARS symptoms. We observed that the average length of time from onset to observed radiographic changes and from onset to first respiratory symptoms were similar (4.4–4.8 days) and generally coincided with admission to hospital. Maximal radiographic changes occurred on the 10th day of illness, on average, 3 days before fever subsided. SARS patients were in hospital for, on average, 24.5 days (± 7.4 days). A total of six (9.7%) case-patients died. We observed that the time from symptom onset to admission decreased during the outbreak (data not shown).

### Case Management

#### Respiratory Therapy

Respiratory assistance was required for 38 (61.2%) of the patients: 25 (40.3%) patients required the use of supplemental oxygen; 6 (9.7%) required positive pressure noninvasive ventilation while an additional 7 (11.3%) patients were intubated and received mechanical ventilation. Only 1 of the 7 who were intubated recovered.

### Antibiotics

A wide range of antibiotics were prescribed for SARS patients in Vietnam, including beta-lactams, tetracyclines, aminoglycosides, macrolides, and fluoroquinolones. Antibiotic therapy was not observed to be clinically beneficial.

### Antivirals

Patients in the first wave of the outbreak of SARS were initially treated with oseltamivir when the etiologic agent was thought to be an influenza virus. Eighteen patients (29.0%) received oral or intravenous ribavirin for an average of 9 days (median 12 days) after the onset of illness. Neither oseltamivir nor ribavirin was observed to have any clinically beneficial effect on the course of illness.

### Steroids

For 14 patients, steroid treatment was begun an average of 8.2 days after the onset of illness (median 7 days). Patients were given steroids for a mean duration of 7.6 days (range 1–14 days). No particular protocol existed for the timing or dosage of steroids given, making interpretation of effectiveness difficult.

## Discussion

This is the first report of a complete outbreak of SARS and as such includes all patients in whom SARS was diagnosed from the beginning of the outbreak until SARS was declared contained in Vietnam on the April 28, 2003. Dr Carlo Urbani (deceased), a public health physician with WHO in Vietnam, first described the outbreak in reports to WHO at the beginning of March 2003. He reported a similar presentation of case-patients that we describe. The main clinical features of probable SARS case-patients reported in Vietnam were fever, malaise, dry cough, and infiltrates on radiographs. These findings are consistent with those reported in Hong Kong (*3*,*7*), Singapore (*8*), and Canada (*9*). Additionally, we have described the clinical development of SARS over time. The main feature exhibited by SARS case-patients on hospital admission was fever, which typically lasted 13–14 days after onset.

Lymphopenia was constant throughout the illness and thrombocytopenia, on average, lasted for 5 days, beginning on the fourth day after onset. Respiratory symptoms and the first radiographic changes were first noted on day 4 of the illness. Maximal radiograph change generally occurred on day 10.

On admission, 6.5% of patients reported having diarrhea. However, patients with SARS may have recalled respiratory symptoms more frequently than gastrointestinal symptoms. During the full course of illness, half of the probable SARS case-patients reported diarrhea. What proportion of these patients had diarrhea directly related to SARS or in response to antibiotic treatment is not known. Diarrhea, regardless of its cause, has important implications for transmission of SARS, because SARS-CoV can be shed in feces (*10*). However, it is not yet known whether viable organisms are shed in quantities sufficient to constitute a substantial source for transmission. The role of diarrhea in SARS transmission requires further investigation.

Our data on admission may not be generalizeable to other SARS outbreaks for several reasons: Admission bias may have occurred at hospital A after the initial cluster among healthcare workers was recognized. In some instances, temperatures were being taken and some patients were admitted after fever onset but no other symptoms, daily chest x-rays were taken for some case-patients, and some patients refused admission until after they had been ill for several days.

Microbiologic evaluation of patients who met the case definition for probable SARS in Vietnam was difficult at the time of admission. Decisions about case status on admission were initially made by considering clinical signs and symptoms. We did not have laboratory facilities to confirm SARS, and facilities to identify other agents causing atypical pneumonia were limited. Patients were treated with antibiotics for atypical bacterial pneumonia on admission to hospital, and if the patients responded to treatment within 48 hours, the SARS case status was revised.

All case-patients with probable SARS in the Vietnam outbreak were epidemiologically linked, and 98.4% had serologic evidence of SARS-CoV infection. After the initial case, all probable SARS cases identified in the Vietnam outbreak were among healthcare workers or close contacts of case-patients.

Our findings in regard to treatment are nonspecific. Proven treatment options must await proper clinical trials in other centers.

Despite the nonspecific nature of SARS at clinical presentation, a typical case had fever, myalgia, malaise followed several days later by cough and respiratory symptoms. At this point the patient typically had changes shown by chest x-ray, lymphopenia, and thrombocytopenia. Due to the nonspecific nature of SARS, both on admission and throughout the course of illness, clinicians must obtain a detailed exposure history for anyone presenting with atypical pneumonia to help in the early diagnosis and management of a potential outbreak situation. When the diagnosis is in doubt, the person should be isolated under strict infection control procedures until the diagnosis becomes clear.

## References

[1] Centers for Disease Control and Prevention. Update: Outbreak of severe acute respiratory syndrome—worldwide, 2003. Morb Mortal Wkly Rep MMWR. 2003;52:241–8. 12680518

[2] World Health Organization. Cumulative number of reported probable cases of severe acute respiratory syndrome (SARS). URL: http://www.who.int/csr/sars/country/table2003_09_23/en/

[3] Peiris JSM, Lai ST, Poon LLM, Guan G, Yam LY, Lim W, Coronavirus as possible cause of severe acute respiratory syndrome. Lancet. 2003;361:1319–25. 10.1016/S0140-6736(03)13077-2 12711465 PMC7112372

[4] Ksiazek TG, Erdman D, Goldsmith CS, Zaki SR, Peret T, Emery S, A novel coronavirus associated with severe acute respiratory syndrome. N Engl J Med. 2003;348:1953–66. 10.1056/NEJMoa030781 12690092

[5] Drosten C, Gunther S, Preiser W, van der Werf S, Brodt HR, Becker S, Identification of a novel coronavirus in patients with severe acute respiratory syndrome. N Engl J Med 2003: May 15;348:1967–76. 10.1056/NEJMoa030747 12690091

[6] Global surveillance for severe acute respiratory syndrome. Wkly Epidemiol Rec. 2003;78:97–120. 12723282

[7] Lee N, Hui D, Wu A, Chan P, Cameron P, Joynt GM, A major outbreak of severe acute respiratory syndrome in Hong Kong. N Engl J Med. 2003;348:1986–94. 10.1056/NEJMoa030685 12682352

[8] Hsu L-Y, Le C-C, Green JA, Ang B, Patton NI, Lee L, Severe acute respiratory syndrome (SARS) in Singapore: clinical features of index patient and initial contacts. Emerg Infect Dis. 2003;9:713–7. 12781012 10.3201/eid0906.030264 PMC3000162

[9] Poutanen SM, Low DE, Henry B, Finkelstein S, Rose D, Green K, Identification of severe acute respiratory syndrome in Canada. N Engl J Med. 2003;348:1995–2005. 10.1056/NEJMoa030634 12671061

[10] World Health Organization. Severe acute respiratory syndrome—multi-country outbreak-update 47: Studies of SARS virus survival, situation in China. [cited 2003 May 7] Available from: URL: http://www.who.int/csr/don/2003_05_05/en/

